# Trustworthy AI: responses to commentators

**DOI:** 10.1007/s44204-024-00229-9

**Published:** 2024-12-23

**Authors:** Christoph Kelp, Mona Simion

**Affiliations:** COGITO Epistemology Research Centre, University of Glasgow, 67-69 Oakfield Ave, Glasgow, G12 8LP UK

**Keywords:** Trust, Trustworthiness, Artificial intelligence, Obligations, Responsibility

## Abstract

In ‘Trustworthy Artificial Intelligence’, we develop a novel account of how it is that AI can be trustworthy and what it takes for an AI to be trustworthy. In this paper, we respond to a suite of recent comments on this account, due to J. Adam Carter, Dong-yong Choi, Rune Nyrup, and Fei Song. We would like to thank all four for their thoughtful engagement with our work, as well as the Asian Journal of Philosophy for publishing the symposium on our paper. The game plan for the paper is as follows. We will first briefly rehearse the account and then respond to comments in turn.

## Trustworthy AI recap

We take the lead from a general account of trustworthiness that we developed in earlier work (Kelp & Simion, 2023). The central idea here is that trustworthiness has to do with being disposed to live up to one’s obligations. Now, we can distinguish between trustworthiness to Φ and trustworthiness simpliciter. Trustworthiness to Φ has to do with being disposed to living up to one’s obligations to Φ. For instance, to be trustworthy to pick up the kids from school is to be disposed to live up to one’s obligations to pick up the kids from school. On the other hand, trustworthiness simpliciter has to do with being disposed to living up to one’s obligation simpliciter.

On the face of it, this account seems particularly ill poised to explain how it is that AI can be trustworthy and what it takes for an AI to be trustworthy. After all, it might be thought that AIs simply do not have obligations. On closer inspection, however, there is excellent reason to think that appearances are misleading here. In short, this is because AIs have functions that have normative import. In other words, as a result of having functions, there are facts about what AIs should do. By way of illustration, take a paradigm example of a functional item: the human heart. The human heart has the function of pumping blood by beating at a certain range when hooked up to arteries and veins in a certain way. As a result of having this function, there are facts about how the heart should function; to wit, it should do so by beating at a certain rate (normal functioning) and it should pump blood (function fulfilment) when beating at a certain rate (normal functioning) and hooked up to arteries and veins in a certain way (normal conditions) (e.g. Millikan, 1984).

Crucially, since AIs have functions, there are facts about how they should work. In this way, our account can explain how it can be that AIs can be trustworthy. What’s more, we can also now see what it takes for AIs to be trustworthy. AIs may be more or less disposed to do the things that they have the function of doing and to do them in the way that they were designed or selected to, and that is what it takes for them to be more or less trustworthy.

## Song

Song (2023) argues that the goodwill view compares favourably with our view and develops a version of the goodwill account that can capture the motivations of our account.

In her comparison between the goodwill account and our account, Song first points out that the claim that there is trustworthy AI is controversial and that it has been denied in the literature. What’s more, while the goodwill account may not allow us to classify any current AI as trustworthy, it is not incompatible with the existence of trustworthy AI. In particular, it can allow that there may well be trustworthy AI in the future (when AI has the right kind of agency and good will). In light of this, Song takes it to be not entirely clear that the data favour our account over the goodwill account (2023, 2–3).

What’s more, Song also argues that our account faces a problem that arises from the distinction between trust and reliance. Song follows the standard route in the literature of unpacking this distinction in terms of vulnerability to betrayal: trust but not reliance can be betrayed (e.g. Baier, 1986). The problem for our account then is that it has the untoward consequence that we can be betrayed by AIs (2023, 3).

In light of this, Song embraces the consequence of the goodwill account that current AI can only be reliable, not trustworthy. Nonetheless, she sketches a positive account that is aimed at accommodating the central motivations of our view. To begin with, Song observes that AIs are embedded in networks with several constituent parts, including some that possess goodwill and so can be trustworthy. Her central idea is that we can and should trust these networks if the constituent parts that do possess goodwill are trustworthy and the ones that do not are reliable. While current AIs may not be trustworthy, the motivations for assessing them as trustworthy can be captured by allowing that we can and should trust various networks that they are constituent parts of (2023, 4–5).

As a first observation by way of response, the dialectical situation in the debate is delicate. True, the existence of trustworthy AI has been denied in the literature. However, we are not convinced that this point carries much weight. After all, this is exactly what prominent accounts such as the goodwill account entail. The worry here is that any controversy surrounding the existence of trustworthy AI is rooted in the support that accounts such as the goodwill account enjoy. If so, the fact that the existence of trustworthy AI has been denied in the literature cannot be used to defeat reasons against accounts that predict that there is no trustworthy AI, at least not without further ado.

What about the undesirable consequence that our account is said to have, i.e. that we can be betrayed by AIs? First, let us try and reconstruct this argument as well as we can, to give it the most charitable read. It would seem as though the thought would roughly go along the following lines: trust implies the possibility of betrayal; therefore, the impossibility of betrayal implies the impossibility of trust; trustworthiness implies the permissibility of trust; permissibility implies possibility; as such, since our account of trustworthiness implies that trusting AI can be permissible, it implies that trusting AI can be possible, and thereby that being betrayed by AI is possible.

A few of things about this. First, it’s not clear to us that the conclusion that our account implies that AI can betray us follows as straightforwardly as assumed, since the line of reasoning above is controversial at a few junctures: most^1^ importantly, it is straightforwardly false that trustworthiness implies the permissibility of trust: one can be impeccably trustworthy while, at the same time, be the victim of a jokester that fills the environment with misleading evidence that they are maximally untrustworthy. In a case like this, it is not permissible for anybody to trust them, although they are trustworthy. Trustworthiness does not imply permissible trust, just like truth does not imply justified belief.

Third, it is not clear to us at all that it is counterintuitive that we can be betrayed by AIs. One way not to settle this issue through a mere battle of intuitions would be to get clearer on what this possibility of betrayal (supposedly implied by trust) maps on to, and, even more importantly, how it differs from a mere possibility of being disappointed (supposedly corresponding to mere reliance). We really like a case by Katherine Hawley that illustrates this difference: the thought is that, just because your colleague John brings extra sandwiches to work every day, you are not thereby entitled to trust him to do so — since you’re not entitled to feel betrayed if he does not; compatibly, given a large inductive basis, you may be entitled to rely on him to do so, and be disappointed if one day he does not bring any sandwiches (2019, 3). The case suggests that the relevant difference is one in type of expectation: you are entitled to expect in the predictive sense (warranted by e.g. induction) that John will bring sandwiches, but you’re not entitled to expect in the normative sense (warranted by the existence of some norms that impose this duty on John) that John will bring sandwiches.

But now note that, if that is the relevant difference between entitled trust and entitled reliance — i.e. the type of expectation that they map on to — then any account should be able to predict that AI can betray us, since AI is governed by norms sourced in its functioning, and which warrants normative expectations. Furthermore, this is not only perfectly compatible with our account, but also predicted by it. After all, on our view, trustworthiness, just like warrant for normative expectation, has to do with obligation fulfilment.

On the other hand, suppose that it is indeed impossible to be betrayed by AIs, artefacts and the like. If so, on the betrayal account, it is impossible to trust AIs, artefacts and the like. Crucially, this flies in the face of our ordinary conception of trust. We take ourselves to trust artefacts, AIs, etc. as a matter of course. For instance, we take ourselves to trust our car (or self-driving car if we want to think of AIs) because it is a trustworthy brand, well maintained, almost new, etc. In contrast, we take ourselves to distrust our neighbour’s car because it is an old, tattery vehicle that has not passed a vehicle safety test in years. According to any version of the betrayal account on which it is impossible to be betrayed by AIs, artefacts and the like, we are mistaken in taking ourselves to trust/distrust cars. What we really do is rely on our car but not the neighbour’s. Any such version of the betrayal account will need an error theory for our ordinary conception of trust and our practice of attributing trust in AIs, artefacts, etc. And, if anything, that is a strike against any such view.^2^

Finally, the fact that goodwill accounts are in principle compatible with trustworthy AI does not help alleviate the worry for accounts such as the goodwill account, at least not so long as these accounts entail that current AIs do not have what it takes to be trustworthy. We are at a point in time at which it is a desideratum on accounts of trustworthiness that AIs can be trustworthy. To see this, note, for instance, that we live in an age in which AIs are much better at diagnosing certain forms of cancer than human doctors. If people do not trust these diagnostic AIs, this will be an obstacle to the wellbeing of individuals and the population at large. What we need is an account of trustworthiness that allows AIs to be trustworthy, because this will allow us to explain how placing trust in AIs can be rational and may allow us to take steps towards getting people to trust AIs. The point that goodwill accounts can allow for trustworthy AIs at some point in the future, when AIs have wills, simply does not help with addressing this problem. (It may also be worth noting that it will not help to respond that while we cannot trust these AIs, we can rely on them. The reason for this is simply that people may well be unwilling to rely on AIs when they do not trust them.) In light of these considerations, we take it that the fact that our account allows for trustworthy AI favours our view over the goodwill account.

What about Song’s alternative view? Recall that her central idea is that we can and should trust the networks she countenances if the constituent parts that do possess goodwill are trustworthy and the ones that do not are reliable. We can see at least two problems with this view.

The first is that the view does not really succeed in improving on ours when it comes to overpredicting the possibility of betrayal. Since on Song’s view we can trust networks, the betrayal view delivers the result that we can be betrayed by networks. However, that seems just as plausible/implausible as the claim that we can be betrayed by AIs. Our second worry concerns Song’s claim that if the constituent parts that do possess goodwill are trustworthy and the ones that do not are reliable, we can and should trust these networks. In short, the reason for this is that networks may inflict systemic injustices against certain individuals or groups (think of systemic racism or sexism), even if all constituent parts of the network are operating with perfect goodwill/reliability. Song’s account predicts that we can and should trust such networks. Crucially, however, we take it that this is the wrong result.

## Choi

Choi (2023) raises two worries for our view. The first has to do with supererogation, the second with the normative import of functions. We will start by looking at his concern about supererogation.

Choi’s concern is that, all else equal, someone, A, who is disposed to supererogate is more trustworthy than someone, B, who is not. However, Choi argues, our account cannot accommodate this datum. After all, since all else is equal between A and B, they are both equally disposed to fulfil their obligations. Accordingly, our account predicts that A and B are equally trustworthy (2023, 4–5).

By way of response, we would like to introduce a standard distinction in the literature on supererogation between three approaches to this phenomenon: antisupererogationism, qualified supererogationism, and unqualified supererogationism (Heyd, 2024, §3). Crucially, two out of these three approaches countenance an obligation to perform the supererogatory act. Anti-supererogationism (e.g. Feldman, 1986; Moore, 1903) simply denies the existence of supererogatory acts and takes them to be obligatory instead. Qualified supererogationism (e.g. Cohen, 2013; Raz, 1975) also countenances an obligation but adds that it makes no sense to enforce them/that we have an excuse for not complying with them/that there is a higher-order permission not to act on the reasons provided by the supererogation. It is easy enough to see that, on both views, there is no problem for the account. If A is disposed to supererogate and B is not, then A is more strongly disposed to fulfil their obligations than B.

The only approach, if any, that may cause trouble for our view is unqualified supererogationism (e.g. Crisp, 2013; Stangl, 2016), which does not countenance an obligation to perform the supererogatory act. Two quick points on this: First, we are not attracted to this kind of view. The main reason for this is that we would like to preserve value-norm links such as ‘we ought to promote value’. Unfortunately, we do not have the time and space to elaborate on this here (but see e.g. Simion, 2021; Kelp & Simion, 2021).

Instead and second, we will point out that, at the very least, we are siding with the majority here. By way of evidence, note that the current SEP entry on supererogation takes qualified supererogationism to be ‘most common in the literature on supererogation’ (§3.2). We take it that this will at least suffice to shift the burden of proof back onto Choi’s shoulders. In order to make this point stick, he’ll first have to make a case for unqualified supererogationism.

Third, it is not clear to us that supererogation cases — even read as unqualified — are as problematic as Choi suggests to begin with. Here is why: first, it seems that, intuitively, not all cases of supererogation will be trouble: consider a case in which we have to choose between taking our kids to nursery A and nursery B. A and B have identical track records in all respects. The only difference is that B gives the kids a sticker at the end of the week. Our intuition is as follows: A and B are equally trustworthy nurseries. Compatibly, the slight difference in value makes it OK to prefer B over A. But this is exactly the result predicted by our account — supererogation does not make a difference to trustworthiness.

What kind of (alleged) cases of supererogation make trouble for our account? We want to say: non-genuine cases of supererogation. Say, for instance, that in the case above, nursery B has an identical track record to nursery A in all respects, with the exception of the fact that they serve more fruit for dessert. Say, also, that both A and B are impeccably meeting the laws governing childcare. Just as in the case above, it makes sense to prefer nursery B to nursery A. Unlike in the case above, however, here, one may get an intuition that the choice-worthiness of B lies with its superior trustworthiness, which, in turn, lies with its supererogatory behaviour: We agree that one should prefer B to A, and that this is (at least partially) explained by B being more trustworthy than A. We disagree that this is a genuine case of supererogation; however, after all, nurseries are not only governed by laws, but they are also governed by moral and social norms. Giving kids very healthy meals certainly features among these norms.

Can’t we stipulate that B is overdoing the healthy provision, though? We can, but then the intuition of superior trustworthiness (and maybe even that of choice-worthiness) threatens to disappear: do we really prefer a nursery that gives my kids one too many apples? This is not clear at all.

In a nutshell: we do not mind supererogation. It either is unpacked in terms of obligation — in which case it sits nicely with our account — or it is not, in which case it is not clear that it creates any trouble at all.

Choi’s second worry concerns the normative import of functions. He writes:

The function account assumes that if an AI’s function is an etiological function or design function, then that AI is obliged to fulfill the function and follow norms which are conducive to fulfilling the function. However, from the fact that an AI has certain etiological or design functions, it does not follow that the AI is obliged to fulfill the functions and follow relevant norms. (2023, 6)

Choi also considers a range of possible responses on our behalf and finds them either wanting or unavailable to us.

In response, pace Choi, it is not part of the normative import of functions that functional items have an obligation to fulfil their functions. To see this, consider a standard GPS for cars. Its function is to guide people to their destinations by mapping out an accurate route to the destination. Now, it may be that this particular GPS is never installed in a car and so never fulfils its function. This does not mean that it fails to fulfil its obligations. The reason for this is that there are qualifications on the normative import of functional items: What ought to be the case is that they fulfil their function *when functioning normally in normal conditions*. In the case of the GPS that is never installed, the GPS is not in normal conditions. We can also consider a GPS that is installed in a car that is never used. Unlike the first GPS, this one is in normal conditions, but it is never functioning, and so not functioning normally. In both cases, the GPS does not violate any of the obligations that it has in virtue of having a function.

## Carter (and Nyrup)

Carter’s (2023) worry is that our account faces a dilemma. The crux of it is that the functions that generate obligations can either be restricted to representational functions or they can include non-representational functions as well. Both options are unattractive. If so, the account is in trouble (2023, 6).

Let us start with the first horn according to which only representational functions generate obligations relevant to trustworthiness. If so, then AIs that do not have representational functions are not classified as trustworthy or untrustworthy. However, that is plainly implausible. There are AIs that do not have representational functions that we still want to be able to classify as trustworthy. Among the examples of AIs that do not have representational functions Carter mentions are Alpha-Code and YouTube’s recommender system (YRS). In the case of Alpha Code, this is because it is optimised for practically useful coding, and in the case of YRS, it is because it is optimised to keep people watching. If we embrace this horn, we would have to agree that Alpha-Code, YRS and many other AIs that do not have representational functions fail to satisfy the conditions for trustworthy AI (2023, 5).

We agree with Carter that this would be an undesirable result. In light of this, let us look at the second horn. The trouble with this horn, according to Carter, is that our account is too permissive. For instance, it might be that YRS is maximally disposed to comply with all of its function-generated obligations and still widely promotes conspiracy theories in users. In that case, our account predicts that YRS is maximally trustworthy. However, that seems to be the wrong result. After all, anything that widely promotes conspiracy theories is not trustworthy at all (2023, 6).

Before we move on, we would like to note that the same kind of worry has also been raised in Nyrup’s (2023) contribution. Whereas Carter focuses on stating the dilemma, Nyrup focuses on the second horn (2023, 4–6) and sketches an alternative account of trustworthy AI, which is aimed to improve on our view. For now, we will focus on responding to the objection. We will discuss the alternative proposal in the next section.

As a first observation, note that our account of trustworthy AI is in the first instance an account of trustworthiness to Φ for AI, where the relevant Φ-ings are the functional effects of AIs. What our account predicts about YRS then is that it is trustworthy when it comes to keeping people watching insofar as it is disposed to fulfil its function of keeping people watching when functioning normally in normal conditions and insofar as it is disposed to function normally. We take it that this is entirely as it should be. In particular, if YRS is maximally disposed to keep people watching when functioning normally in normal conditions, then YRS is indeed maximally trustworthy when it comes to keeping people watching. Note also that our account predicts that YRS is not trustworthy when it comes to such things as preventing conspiracy theorising and doing the morally right thing. After all, YRS does not have any relevant functions and so no associated obligations. Trivially, then, YRS is not disposed to comply with these norms.

While we do focus on trustworthiness to Φ in the paper, one may wonder about trustworthiness simpliciter when it comes to AI. What’s more, it might be thought that the kind of objection that Carter and Nyrup press is best understood as an objection to trustworthiness simpliciter. After all, even if we want to allow that YRS can be maximally trustworthy when it comes to keeping people watching, we might not want to say that YRS is maximally trustworthy simpliciter. But, on our account, it is hard to see why we should not say this. After all, YRS may well be maximally disposed to satisfy all the obligations it has.

In response, we would first like to flag a distinction that we develop in more detail in the general paper on trustworthiness and only briefly touch upon in the paper on trustworthy AI. This is the distinction between attributive and predicative trustworthiness simpliciter. Attributive trustworthiness explicitly specifies a domain of attribution as in ‘George is a trustworthy babysitter’. In contrast, predicative trustworthiness, e.g. ‘George is trustworthy’, does not. Now, if we want our account to extend beyond trustworthiness to Φ, the natural target is attributive trustworthiness. This is already suggested by the title, which is, after all ‘Trustworthy Artificial Intelligence’. But now, note that when it comes to attributive trustworthiness, there is again reason to think that the predictions of our account are entirely correct. To see this, note that one can be a trustworthy gang member, say, even though the things one does are much worse than promoting conspiracy theorising in the population. If one’s kingpin orders a hit, then the fact that one is disposed to carry it out swiftly counts towards one’s being a trustworthy gang member. This is notwithstanding the fact that what one is doing is morally abhorrent. And the same goes, mutatis mutandis, for trustworthy AI.

## Nyrup

We already mentioned that Nyrup has similar worries for our account as Carter. However, he also sketches an alternative, which we will look at now. Nyrup agrees with our general view that unpacks trustworthiness in terms of a disposition to fulfil one’s obligations. Nyrup’s key idea is, in essence, that these obligations are sourced in the norms that should govern the social practices that AIs participate in. Here, part of what it takes for it to be true that certain norms ‘should govern’ a social practice is that they would best allow the practice to achieve its overall social function (2023, 7–8).

One may wonder whether this helps. Consider YRS. Its social function is to recommend watchable content. And it may well be that it does so perfectly. If so, then Nyrup’s account does not appear to improve on ours on this front. After all, while doing so perfectly, it may still promote conspiracy theories. Nyrup recognises this problem, or at least a version of it. He grants that social practices may have unjust ends and acknowledges that we might not want to call AIs that serve unjust ends trustworthy. His suggestion, which is only briefly hinted at towards the end of the paper, is to go for what he calls a ‘normative theory’ of social practices, according to which they are defined ‘as patterns of learned behaviour that allow agents to coordinate their actions in ways that benefit everyone or that allow them to hold each other appropriately accountable’ (2023, 9). This also will not work. If social practices are inherently normative in this way, then the practice involving YRS threatens simply not to be a social practice.

An improvement might be that social practices are governed by moral norms and that AIs are subject to moral norms as a result of participating in these social practices. While this may deliver the result that YRS is not trustworthy because it violates these moral norms, we are also wary of this solution. Lots of things participate in social practices, including various artefacts that are not AIs and natural kinds. But surely, we would not want to say that moral norms apply to all sorts of artefacts and natural kinds just because they participate in social practices. Now, Nyrup is clear that the reason why he takes AIs to have obligations in virtue of participating in social practices is that they play human-like roles in these practices (2023, 7). However, even this seems to overgenerate moral norms. Lots of artefacts play human-like roles. Just think of the mechanisation of (the social practice of) manufacturing. It strikes us as implausible that artefacts that have replaced humans in the production of goods are subject to moral norms because they play human-like roles in some social practices.

Since we have seen that there is reason to think that our account of trustworthy AI gives the right verdict even in cases of YRS and since there is reason to resist Nyrup’s alternative, we take it that we will do well to hold on to our account of trustworthy AI. Before closing, it may be worth noting that we can accommodate the intuition that Nyrup registers. After all, we can agree with Nyrup that there are moral norms governing social practices. What’s more, these moral norms govern how we should use AIs in certain social practices, whether we should use them at all in others, whether we should modify them, etc. For instance, the fact that YRS promotes conspiracy theorising is a reason to use it with caution or perhaps not at all, to modify its mode of operation, etc. Crucially, however, while there are moral norms governing our use of YRS, they do not bear on how trustworthy an AI such as YRS is.^3^

## Data Availability

Not applicable.

## Footnotes

1 There’s also a question concerning the fact that while many formal models will predict it, it is not clear at all that it is analytic that permissibility implies possibility. Is it impermissible to be a round square? We do not know.

2 It may be worth noting that there are further reasons to think that the betrayal account is ultimately unsatisfactory. Consider the US’s official motto is ‘In god we trust’. There can be little doubt that trust in god is compatible with god being such that there is no risk of betrayal by god, say because god’s omnipotent, omniscient, and omnibenevolent nature excludes any such risk. If so, there is reason to think that trust does not require vulnerability to betrayal (Kelp & Simion 2023).

3 We do not analyse or generate any datasets, because our work proceeds within a theoretical and mathematical approach. Ethical approval is not applicable for this research. The research for this paper was supported by grants from the Leverhulme Trust (Grant/Award Number: RPG-2019-302) and the European Research Council (Grant/Award Number: 948356).
